# Stable Water Use Efficiency under Climate Change of Three Sympatric Conifer Species at the Alpine Treeline

**DOI:** 10.3389/fpls.2016.00799

**Published:** 2016-06-07

**Authors:** Gerhard Wieser, Walter Oberhuber, Andreas Gruber, Marco Leo, Rainer Matyssek, Thorsten Erhard Edgar Grams

**Affiliations:** ^1^Department of Alpine Timberline Ecophysiology, Federal Research and Training Centre for Forests, Natural Hazards and LandscapeInnsbruck, Austria; ^2^Institute of Botany, Leopold-Franzens-Universität InnsbruckInnsbruck, Austria; ^3^Ecophysiology of Plants, Department of Ecology and Ecosystem Management, Technische Universität MünchenFreising, Germany

**Keywords:** stable isotopes, intrinsic water use efficiency, tree growth, climate change, treeline, Central Alps

## Abstract

The ability of treeline associated conifers in the Central Alps to cope with recent climate warming and increasing CO_2_ concentration is still poorly understood. We determined tree ring stable carbon and oxygen isotope ratios of *Pinus cembra, Picea abies*, and *Larix decidua* trees from 1975 to 2010. Stable isotope ratios were compared with leaf level gas exchange measurements carried out *in situ* between 1979 and 2007. Results indicate that tree ring derived intrinsic water-use efficiency (iWUE) of *P. cembra, P. abies* and *L. decidua* remained constant during the last 36 years despite climate warming and rising atmospheric CO_2._ Temporal patterns in *Δ^13^C* and *Δ^18^O* mirrored leaf level gas exchange assessments, suggesting parallel increases of CO_2_-fixation and stomatal conductance of treeline conifer species. As at the study site soil water availability was not a limiting factor iWUE remained largely stable throughout the study period. The stability in iWUE was accompanied by an increase in basal area increment (BAI) suggesting that treeline trees benefit from both recent climate warming and CO_2_ fertilization. Finally, our results suggest that iWUE may not change species composition at treeline in the Austrian Alps due to similar ecophysiological responses to climatic changes of the three sympatric study species.

## Introduction

High-altitude forest ecosystems at the timberline-treeline transition have raised concern as they may undergo significant alterations due to climate warming and changes in ground-level air chemistry (Holtmeier and Broll, 2007; Wieser et al., 2009). Dendroclimatological studies conducted within the treeline ecotone of the Central European Alps have shown radial stem growth to be limited by low summer temperature (Carrer and Urbinati, 2004; Oberhuber, 2004; Büntgen et al., 2005; Frank and Esper, 2005; Oberhuber et al., 2008). During recent decades, several authors report treeline-associated conifers to reflect increased radial growth, putatively related to climate warming (Graumlich et al., 1989; Peterson et al., 1990; Jacoby and D’Arrigo, 1997; Rolland and Florence-Schueller, 1998; Bunn et al., 2005). Moreover, increasing atmospheric CO_2_ concentration may act in concert with climate warming to increase carbon accumulation within the treeline ecotone (cf. Graumlich, 1991; Saurer et al., 1997; Duquesnay et al., 1998; Sidorova et al., 2009). Notwithstanding, reduced radial growth has been attributed to late-summer drought under increasing treeline temperature in the European Alps (Büntgen et al., 2006; Carrer and Urbinati, 2006; Oberhuber et al., 2008; Wieser et al., 2009).

Stable isotope ratios of carbon and oxygen, i.e., ^13^C/^12^C and ^18^O/^16^O, respectively, may serve as dendrochronological proxies that facilitate mechanistic understanding of climate-related influences on physiological processes such as leaf gas exchange and stem wood formation (Loader et al., 2007; Weigt et al., 2015; and further references therein). In plant organic matter, *δ^13^C* expressing the ^13^C/^12^C ratio in relation to an international standard (Pee Dee Belimnite) depends on variables such as leaf conductance for water vapor (*g*_w_) that modify the net CO_2_ uptake rate (*A*; Farquhar et al., 1989). In addition, *δ^13^C* of plant organic matter (δ^13^C*_p_*) is a function of atmospheric *δ^13^C*, which is accounted for by calculating discrimination of photosynthesis against ^13^C (*Δ^13^C;* Farquhar and Richards, 1984) in relation to intrinsic water-use efficiency (iWUE), i.e., the ratio of *A* (rate of net CO_2_ fixation) versus *g*_w_ (leaf conductance for water vapor). For a review see Brugnoli and Farquhar (2000).

When analyzing *δ^13^C_p_* alone, the impacts of *A* (demand of CO_2_) and *g*_w_ (supply of CO_2_) on iWUE are difficult to separate (Saurer et al., 2008). The oxygen isotope ratio *(δ^18^O*), however, may allow a distinction between biochemical and stomatal limitations of photosynthesis as it is not affected by the photosynthetic CO_2_ carboxylation but linked to g_w_ (Barbour et al., 2000; Grams et al., 2007). It is, therefore, an ideal covariable to estimate to what degree photosynthesis and stomatal conductance modify *δ^13^C_p_* (Scheidegger et al., 2000; Werner et al., 2012). Originally, this dual isotope approach was introduced for photosynthetic tissue and only recently tested conceptually for the interpretation of tree-ring data (Roden and Farquhar, 2012), although several critical points should be taken into account during interpretion. Among the most important issues that need to be considered are the facts that the *δ^18^O* of source and atmospheric water can vary spatially and temporarily and that post-photosynthetic and post-evaporative oxygen atom exchange processes could affect the initial leaf-level isotope signal (see below).

At the leaf level, *δ^18^O* of photoassimilates derive primarily from leaf water, typically being enriched in ^18^O compared to the source water (i.e., xylem water) through evaporative enrichment at the site of transpiration. This enrichment is counteracted by the so-called Péclet effect and transpiratory leaf cooling (for a review see Barbour, 2007), which may result in the above mentioned negative correlation between *δ^18^O_p_* and *g_w_* (e.g., Barbour et al., 2000; Grams et al., 2007). However, to an extent that may depend on species and site conditions, the signal is dampend by oxygen exchange with source water during biomass formation at the stem level (Gessler et al., 2013). This causes, at least partially, a decoupling between oxygen isotopic signatures of photoassimilates and the tree ring organic matter. However, in a recent report Weigt et al. (2015) confirmed that information may be exploited to relate ecophysiological responses of trees to environmental changes for both total sapwood organic matter and extracted cellulose. In any case, it appears advisable to confirm interpretation from the dual isotope approach by gas exchange assessments whenever possible.

Previous tree-ring carbon isotope studies carried out in tropical, arid, Mediterranean, temperate and boreal forest ecosystems have identified an increase in iWUE over the past 40 years in response to increasing atmospheric CO_2_ concentration (Penuelas et al., 2011; Saurer et al., 2014) and just recently supported by tree-ring δ^13^C dynamic global vegetation models comparisons (DGVMs; Frank et al., 2015). Tree growth on the contrary, remained stable or even declined, suggesting local site conditions to override a potential CO_2_-induced increase in growth (Penuelas et al., 2011; Silva and Anand, 2013; Levesque et al., 2014). Increasing iWUE accompanied by a reduced productivity has been attributed to the combined effect of elevated CO_2_ and climate change-induced soil drying (Penuelas et al., 2011; Saurer et al., 2014).

The impact of the steadily increasing CO_2_ level and concurrent climate change, however, still awaits clarification for treeline-associated conifers in the Central Austrian Alps where low temperatures limit tree growth (Oberhuber, 2007; Wieser et al., 2009). At treeline in the Central Austrian Alps ample precipitation during the growing season prevails every third to fourth day on average (Wieser, 2012), so that soil water limitation stays absent, allowing trees to meet their water demand (Tranquillini, 1979; Mayr, 2007; Matyssek et al., 2009). Hence, whole-tree conductance stays high and mainly depends on the evaporative demand in terms of irradiance and vapor pressure deficit (Wieser, 2012). Therefore, we hypothesize that treeline trees passively respond to the increasing atmospheric CO_2_ level (*C_a_*), so that their leaf-intercellular CO_2_ concentration (*C_i_*) rises in parallel, while iWUE remains unchanged. The hypothesis was evaluated by stable carbon and oxygen isotope sampling and radial growth analysis over the past 36 years (1975–2010) in stems of mature *Pinus cembra, Picea abies* and *Larix decidua* trees growing at the treeline of Mt. Patscherkofel in the Central Tyrolean Alps. Observed long-term trends in δ*^13^C_p_, δ^18^O_p_* in tree rings and iWUE were at least for some years compared with *in situ* leaf-level gas exchange data, assessed at the same study site between 1979 and 2007 in adult *P. cembra* and *L. decidua* trees. Results are discussed in view of tree response to climate warming at the treeline ecotone.

## Materials and Methods

### Study Site, Climatic Data, and Tree Species

The study was conducted in a scattered stand at the lower edge of the treeline ecotone at 1950 m a.s.l. on Mt. Patscherkofel (47°12′37″ N, 11°27′07″ E), south of Innsbruck, Austria. The site is characterized by a cool subalpine climate, the possibility of frost during the entire year and a continuous snow cover from October through April. We used monthly mean temperatures and monthly total precipitation from 1975 to 2010 from a weather station nearby (Klimahaus Research Station and Alpengarten; 1950 m a.s.l.) for our analysis. Mean annual precipitation averaged 878 mm, with 58% falling during the growing season (May through September). Mean annual air temperature averaged 2.4°C, with summer maxima of up to 27°C and winter minima of -28°C.

The geology of Mt. Patscherkofel is dominated by gneisses and schist. The soil at the study site is a haplic podzol, being a typical soil type of the treeline ecotone in the Central Tyrolean Alps (Neuwinger, 1972). The water holding capacity of the soil (at 5–65 cm depth) at saturation (-0.001 MPa) averages 0.60 m^3^ m^-3^. Due to frequent precipitation during the growing season soil water potential rarely drops below -0.01 MPa, approximating soil water contents above 0.35 m^3^ m^-3^ (Wieser, 2012; including the dry summer of 2003, unpublished data).

The stand is composed of the dominant tree species *Pinus cembra*, accounting for 84% of the tree population, and accompanied by *Larix decidua* (9%) and *Picea abies* L. Karst (7%) at some locations. Trees grew either as isolated trees or in groups of four to five. The distance between single trees or tree groups was 20–30 m. From 20 *P. cembra* trees cored at the lower edge of the treeline ecotone Oberhuber et al. (2008) derived an expressed signal population (EPS) value of 0.94, reflecting a strong climate signal in the site chronology. From these trees we selected five trees which had the strongest correlation to the site specific mean tree-ring chronology, no missing rings, and regular ring boundaries. In addition we cored five dominant *P. abies* and *L. decidua* trees each to account for potential inter-specific differences of the three associated treeline species. In 2010 the trees were 69 ± 9 years old, with stem heights averaging 12 ± 1.3 m. The stem diameter at breast height (DBH) averaged 22 ± 3.2 cm.

### Tree Ring and Basal Stem Area Increment

In fall 2010 we obtained two increment cores per trees at DBH using a 5-mm-diameter increment bore. For contrast enhancement of tree ring boundaries the cores were dried in the laboratory, non-permanently mounted on a holder, and the surface was prepared with a razor blade (Pilcher, 1990). Ring widths were measured to the nearest 1 μm using a reflecting microscope (Olympus SZ61) and the software package TSAP WIN Scientific. Ring widths of both cores from each sample tree were averaged and individual tree ring chronologies were then checked for dating accuracy using the COFECHA software (Holmes, 1994; Grissino-Mayer, 2001). As ring width may be biased by a negative correlation with the time course during tree maturation, ring width was converted to basal stem area increment (BAI) according to:

(1) $$\text{BAI=3}.\text{14 }(R_{\text{n}}^{2}-R_{\text{n-1}}^{2})$$

where *R* is stem radius inside tree bark and n is the year of tree ring formation (Fritts, 1976). Bark thickness was subtracted from stem radius. Finally BAI of each year were averaged over the five sample trees of each species.

### Stable Isotope Analysis

**FIGURE 1:**
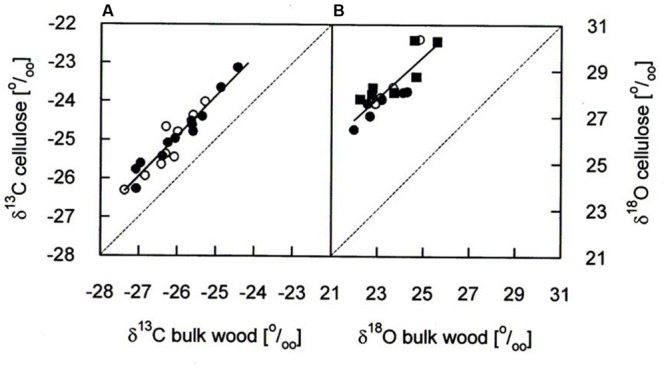
**Comparison of **(A)***δ^13^C* and **(B)***δ^18^O* in bulk wood and cellulose of annual growth rings of *P. cembra* (solid circle), *P. abies* (solid square) and *L. decidua* (open circle).** Dashed lines reflect the one-to-one lines for comparison and the solid lines correspond to the linear regression analyses of the data points: *δ^13^C*: *y* = 1.03x + 1.86, *r^2^*= 0.92, *P* < 0.001, *n* = 36; *δ^18^O*; *y* = 0.92x + 6.65, *r^2^*= 0.73, *P* < 0.001, *n* = 18.

*δ^13^C* and *δ^18^O* analyses for the years 1975–2010 were performed on the same cores as used for BAI assessment. Annual rings (early wood plus late wood) were cut exactly at ring boundaries by use of a scalpel and a reflecting microscope (Wild 308700). For each of the five study trees per species the two samples per tree ring were pooled and homogenized with a swing mill (Retsch MM301, Retsch Haan, Germany). In a subsample, we compared isotope signatures in bulk wood with those in cellulose for determining the necessity of cellulose extraction in our study trees. Cellulose extraction was performed using a modified version of the method of Brendel et al. (2000). The methodological comparison corroborated significant correlations in the cases of *δ^13^C* and *δ^18^O* (**Figure 1**) as reported earlier from coniferous and other tree species (Jaggi et al., 2002; Sohn et al., 2013) and is in accordance with a recent report (Weigt et al., 2015). On average, *δ^13^C* in cellulose was 1.0–1.1 ‰ higher than in bulk wood (**Table 1**), being smaller than 1.3–1.4 ‰ found in *Picea abies* by Borella et al. (1998) and Sohn et al. (2013). Mean *δ^18^O* in cellulose was 4.3–4.9 ‰ higher than in bulk wood (**Table 1**) being somewhat lower than 5.9 ‰ in bulk wood of *Picea abies* (Sohn et al., 2013). Based on these findings and in accordance with a recent methodological study (Weigt et al., 2015), we used bulk wood samples rather than extracted cellulose for isotope analysis.

**Table 1:** *δ^13^C* and *δ^18^O* difference between cellulose and bulk wood in annual growth rings of *Pinus cembra, Picea abies* and *Larix decidua*.

Species	*δ^13^C* [‰]	*δ^18^O* [‰]
*P. cembra*	1.1 ± 0.0	4.4 ± 0.4
*P. abies*	1.0 ± 0.1	5.0 ± 0.6
*L. decidua*	1.1 ± 0.1	4.9 ± 0.4

Regarding *δ^13^C*, 2.0 ± 0.02 mg of homogenized samples were weighed into tin capsules each (3.5 × 5 mm, IVA Analysentechnik e.K., Meerbush, Germany) and combusted to CO_2_ in an elemental analyzer (Eurovector EA3000) connected to an isotope ratio mass spectrometer (Isoprime, Elementar, Hanau, Germany). For *δ^18^O* analysis 0.7 ± 0.05 mg were weighed into silver capsules each (3.5 × 5 mm, IVA Analysentechnik e.K., Meerbush, Germany) to obtain CO at 1,430°C in a high-temperature pyrolysis system (HTO, HekaTech, Wegberg, Germany) which was connected via an open-split interface (Conflo III; Finnigan MAT, Bremen, Germany) to an isotope ratio mass spectrometer (Delta Plus; Finnigan MAT, Bremen, Germany). Isotope abundances were expressed using the *δ*-notation in ‰ relative to the international standards:

(2) $$\delta_{\text{sample}}=(R_{\text{sample}}/R_{\text{standard}}-\text{1})\;*\;1000$$

where *R*_sample_ is the molar fraction of the ^13^C/^12^C or ^18^O/^16^O ratio of the sample, and *R*_standard_ that of the international IAEA standards V-PDB for carbon and V-SMOW for O. The analytical precision was <0.12 ‰ and <0.28 ‰ regarding *δ^13^C* and *δ^18^O*, respectively (expressed as standard deviation of the internal laboratory standard at the same sample mass).

### Isotope Discrimination and iWUE

Tree ring specific *δ^13^C_tring_* were corrected for the progressive decline in atmospheric *δ^13^C_atm_* through calculating ^13^C discrimination (***Δ^13^****C*):

(3) $$\Delta^{13}C(\%)=(\delta^{13}C_{\text{atm}}-\delta^{13}C_{\text{tring}})/(1+\delta^{13}C_{\text{tring}}/1000)$$

To this end, *δ^13^C_atm_* with its nearly linear time course during 1980 through 2010 ^1^ was extrapolated for the years 1975 through 1979. In a simplified model, Farquhar et al. (1982) related *Δ^13^C* through plant physiological processes during CO_2_ fixation in C3 plants with the ratio of intercellular to ambient CO_2_ partial pressure (*C_i_*/*C_a_*):

(4) $$\Delta^{13}C=a+(b-a)*C_{\text{i}}/C_{\text{a}}$$

where *a* (=4.4 ‰) refers to the slower diffusivity of ^13^CO_2_ relative to ^12^CO_2_ in air and *b* (=27 ‰) is the isotopic fractionation caused by enzymatic C fixation. *C_a_* was obtained from published data^2^. It should be noted that *Δ^13^C* is determined by the ratio of chloroplast to the ambient CO_2_ mole fraction (*C_c_/C_a_*) rather than *C_i_/C_a_*, as used in equation 3, making the here calculated value sensitive to mesophyll conductance (*g_m_*; Seibt et al., 2008). The latter varies in accordance to changes in environmental conditions such as temperature, irradiance, water and CO_2_ availability (Flexas et al., 2008). Consequently using *C_a_* may be problematic if *g_m_* to CO_2_ is not constant (Seibt et al., 2008). However, as information on mesophyll conductance of the three conifers under study is not available and published means of *g_m_* would not improve results (Cernusak et al., 2013), we chose using the simplified linear model of Farquhar et al. (1982). Hence, iWUE, i.e., the ratio of the net carbon gain (*A*) *versus* leaf conductance for water vapor (*g*_w_), was calculated as follows:

(5) $$\text{iWUE}=A/g_{\text{w}}=C_{\text{a}}(b-\Delta^{13}C/C_{\text{a}})/1.6*(b-a)$$

where 1.6 is the ratio between the diffusivities of water vapor and CO_2_ in air.

Enrichment in ^18^O in tree rings over source water (*Δ^18^O*), resulting from incorporation of ^18^O-enriched photoassimilates into stem biomass, was calculated from d^18^O of tree ring organic matter (*δ^18^O_tring_*) and precipitation (*δ^18^O_prep_*) according to:

(6) $$\Delta^{18}O(\%)=(\delta^{18}O_{\text{tring}}-\delta^{18}O_{\text{prep}})/(1+\delta^{18}O_{\text{prep}}/1000)$$

Precipitation is the only source of water on Mt. Patscherkofel and thus was assumed to reflect source water of trees. Furthermore, as there is evidence from a treeline in the Central Swiss Alps that mean values of δ*^18^O_prep_* and δ*^18^O* of soil water do not differ significantly, although soil water *δ^18^O* carries a distinct signal from snow melt water far into the growing season (Treydte et al., 2014), we used annual means of δ*^18^*O*_prep_* sampled on top of Mt. Patscherkofel (2246 m a.s.l.) (Umweltbundesamt Austria; personal communication) approximately 300 m south of the selected study trees for our *Δ^18^*O calculation. We elevationally corrected *δ^18^O_prep_*by a factor of 0.17 ‰ per 100 m of elevation (Umweltbundesamt Austria; personal communication) which is considerably lower than the global mean of 0.28 ‰ per 100 m of elevation (Poage and Chamberlain, 2001).

### Leaf Level Gas Exchange Data

To illustrate long term trends in foliar CO_2_ and H_2_O gas exchange we compiled published gas exchange data of mature, field grown *P. cembra* and *L. decidua* trees carried out at our study site between 1979 and 2007 (**Table 2**). Maximum net photosynthetic capacity at ambient CO_2_ (A_max_; sensu Larcher, 2001) was assessed of sun exposed twigs from the upper canopy. Employed *in situ* were thermoelectrically climate-controlled cuvettes (Walz, Effeltrich, Germany) or a portable exchange system (CIRAS 1, PP Systems, Hitchin, Hertfordshire, UK). For methodological details see publications given in **Table 2**.

**Table 2:** A comparison of published maximum net photosynthetic capacity (*A*_max_) and leaf conductance for water vapor of sun exposed shoots of mature *Pinus cembra* and *Larix decidua* trees at the lower end of the treeline ecotone on Mt. Patscherkofel.

Species	Year	Measured trees	*A*_max_ [μmol m^-2^ s^-1^]	*g*_w_ [mmol m^-2^ s^-1^]	Reference
*P. cembra*	1979	1	3.4	n d	Havranek, 1981
*P. cembra*	2002	2	4.6 ± 0.2	n d	Wieser et al., 2005
*P. cembra*	2007	3	5.2 ± 0.7	n d	Wieser et al., 2010
*L. decidua*	1980	1	3.3	48	Benecke et al., 1981
*L. decidua*	1993	4	5.6 ± 0.9	85 ± 14	Volgger, 1995

*n d, not determined.*

### Statistical Analysis

Temperature, precipitation, vapor pressure deficit, *Δ^13^C, Δ^18^O*, BAI, *C_i_, C_i_*/*C_a_* and iWUE trends were calculated for the time period 1975–2010 by least-squares linear regression analysis. For a given variable, differences among trends (slopes) between *P. cembra, P. abies* and *L. decidua* were assessed by the two-slope comparison test (Zar, 1999). We used repeated measures ANOVA to detect significant differences in the mean values (1975–2010) of *Δ^13^C, Δ^18^O*, BAI, *C_i_, C_i_*/*C_a_* and iWUE of *P. cembra, P. abies* and *L. decidua*. Following Kunter et al. (2004) we used multiple least-squares linear regression models to assess the influence of atmospheric CO_2_ concentration (*C_a_*) and mean growing season (May-Sep) air temperature (T_veg_) and their interactions (explanatory variables) on tree-ring variables. For assessing the climatic impact on tree ring variables (BAI, *Δ^13^C*, and *Δ^18^O*) statistical analyses were based on mean monthly air temperature (°C) and total monthly precipitation (mm) throughout the study period (1975–2010). For each species Pearson’s correlation coefficients between BAI, isotope chronologies and both climate variables were calculated from August of the year prior to growth to September of the growth year. All the statistical analysis were conducted by use of the SPSS 16 software package (SPSS. Inc. Chicago, IL, USA**),** and a probability level of *P* < 0.05 was considered as statistically significant. As suggested by Sarris et al. (2013) we did not remove any age related trend from our tree-ring chronologies by conventional detrending procedures, thus avoiding the *risk* of removing any environmental signal or trend captured by our tree-ring series.

## Results

### Inter-annual Trends in Climate and Tree-Ring Indices

**FIGURE 2:**
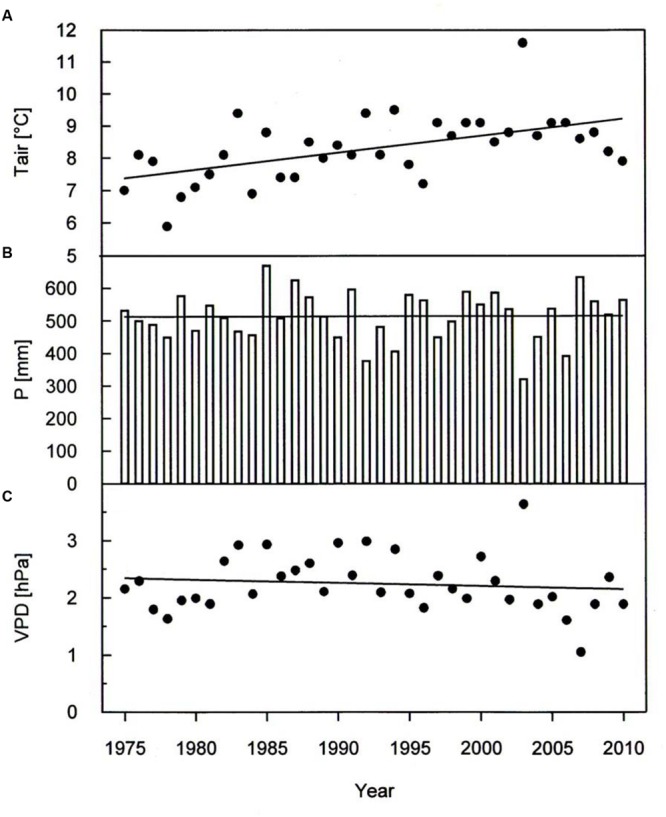
**Temporal variation in **(A)** growing season mean air temperature (Tair), **(B)** total growing season precipitation (P) and **(C)** growing season mean vapor pressure deficit (VPD) during the period 1975 throughout 2010.** Data were fit by linear regression analysis: Tair: *y* = 0.053x-97.0, *r^2^*= 0.30, *P* < 0.001; P: *y* = 0.075x-365.3, *r^2^*= 0.00, *P* = 0.95; VPD: *y* = -0.055x + 13.3, *r^2^*= 0.01, *P* = 0.49.

**FIGURE 3:**
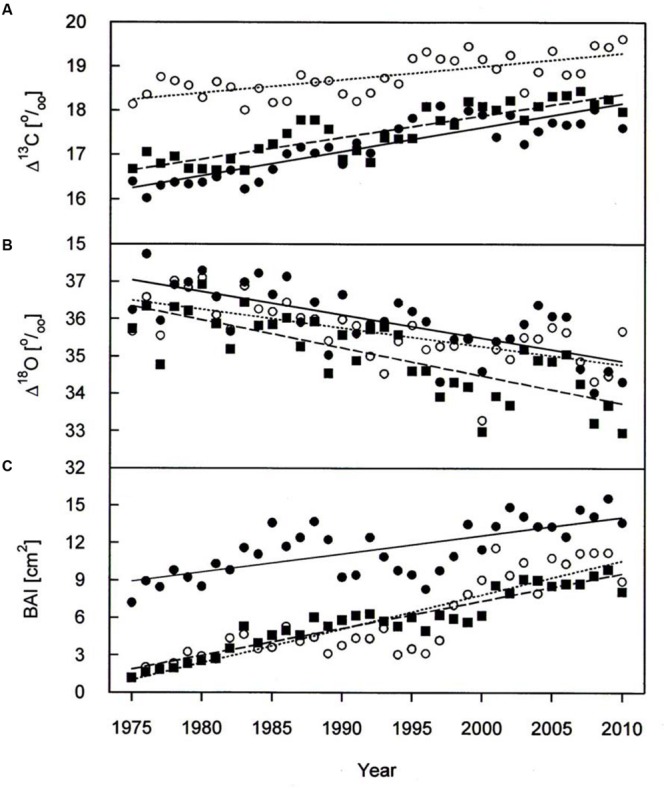
***Δ^13^C***(A)**, *Δ^18^O***(B)**, and BAI **(C)** chronologies of *P. cembra* (solid circle, solid line), *P. abies* (solid square, dashed line) and *L. decidua* (open circle, dotted line) between 1975 and 2010.** See **Table 2** for regression information.

A warming trend is reflected at our treeline site during the growing seasons (0.50°C per decade *P* < 0.001) of 1975–2010, without concurrent trends in precipitation and vapor pressure deficit (**Figure 2**). During the whole study period *Δ^13^C* and *Δ^18^O* chronologies were synchronized between the three studied species. In each species Δ^13^C increased over time (**Figure 3A**; **Table 3**) whereas *Δ^18^O* decline (**Figure 3B**; **Table 3**). The increase in *Δ^13^C* was accompanied by rising of A_max_ for both *P. cembra* (1979–2007) and *L. decidua* (1980–1993) by about 50%. Likewise, *g*_w_ increase by about 75% in *L. decidua* (**Table 3**).

**Table 3:** Regression information for **Figures 3** and **4**.

Variable	Species	Equation	*r^2^*	*P-value*
*Δ^13^C*	*P. cembra*	*y* = 0.054x - 91.3	0.77	<0.001
	*P. abies*	*y* = 0.049x - 79.2	0.76	<0.001
	*L. decidua*	*y* = 0.030x - 40.1	0.49	<0.001
*Δ^18^O*	*P. cembra*	*y* = -0.062x + 159.9	0.50	<0.001
	*P. abies*	*y* = -0.075x + 184.7	0.60	<0.001
	*L. decidua*	*y* = -0.056x + 148.0	0.51	<0.001
BAI	*P. cembra*	*y* = 0.147x - 281.9	0.50	<0.001
	*P. abies*	*y* = 0.219x - 430.8	0.90	<0.001
	*L. decidua*	*y* = 0.273x - 538.4	0.77	<0.001
*C_i_*	*P. cembra*	*y* = 1.81x - 3392.5	0.94	<0.001
	*P. abies*	*y* = 1.75x - 3296.0	0.94	<0.001
	*L. decidua*	*y* = 1.56x - 2873.3	0.94	<0.001
*C_i_*/*C_a_*	*P. cembra*	*y* = 0.002x - 4.33	0.77	<0.001
	*P. abies*	*y* = 0.002x - 3.88	0.76	<0.001
	*L. decidua*	*y* = 0.001x - 2.18	0.62	<0.001
iWUE	*P. cembra*	*y* = -0.085x + 249.4	0.09	0.067
	*P. abies*	*y* = -0.061x + 199.5	0.06	0.148
	*L. decidua*	*y* = 0.045x - 21.9	0.05	0.215

The mean tree-ring *Δ^13^C* was highest in *L. decicua*, although the increase was significantly higher in *P. cembra* and *P. abies* (**Table 4**). Temporal changes in *Δ^18^O* by contrast, did not differ significantly between the tree species (**Table 4**). *P. cembra* showed the highest *Δ^18^O* while *P. abies* presented the lowest *Δ^18^O* and *L. decidua* displayed an intermediate mean (**Table 4**). On average, growth of *P. cembra* was significantly higher than growth of *P. abies* and *L. decidua* (**Figure 3C**; **Table 4**). During the whole study period all three species showed an increase in growth expressed as BAI, being significantly lower in *P. cembra* than *P. abies*, and *L. decidua* (**Figure 3C**; **Table 4**).

**Table 4:** Tree-ring carbon isotope characteristics (*Δ^13^C, Δ*^18^*O*, BAI, *C_i_, C_i_*/*C_a_*, and iWUE) in *P. cembra, P. abies*, and *L. decidua* during the period 1975–2010.

	*P. cembra*		*P. abies*		*L. decidua*	
	Change	Average (±*SE*)	Change	Average (±*SE*)	Change	Average (±*SE*)
*Δ^1^^3^C* [‰]	***1.9***^a^	17.2 ± 0.7^a^	***1.8***^a^	17.3 ± 0.6^a^	**1.1**^b^	18.8 ± 0.484^b^
*Δ^18^O* [‰]	***-2.2***^a^	35.9 ± 0.9^a^	***-2.7***^a^	35.0 ± 1.052^b^	***-2.0***^a^	35.6 ± 0.883^c^
BAI [cm^2^]	***5.3***^a^	7.6 ± 3.8^a^	7.9^b^	5.7 ± 2.473^b^	***9.8***^b^	5.8 ± 3.3^b^
*C_i_* [μmol mol^-1^]	***65.2***^a^	203.5 ± 19.6^a^	***63.06***^a^	208.5 ± 19^a^	***56.2***^a^	228.8 ± 16.9^b^
*C_i_*/*C_a_*	***0.1***^a^	0.6 ± 0.03^a^	***0.1***^a^	0.6 ± 0.03^b^	***0.00***^b^	0.6 ± 0.03^c^
iWUE [μmol mol^-1^]	-3.1^a^	81.0 ± 2.9^a^	-2.2^a^	78.4 ± 2.6^b^	-1.6^a^	67.8 ± 2.2^c^

*Significant changes from 1975–2010 at *P* < 0.05 are in bold and italics. Between species differerences in change and average (±SE) are marked with different letters. Change values were calculated as the slope of the corresponding least-squares linear regressions (**Table 2**) multiplied by the number of years of the study period 1975–2010 (=36).*

**FIGURE 4:**
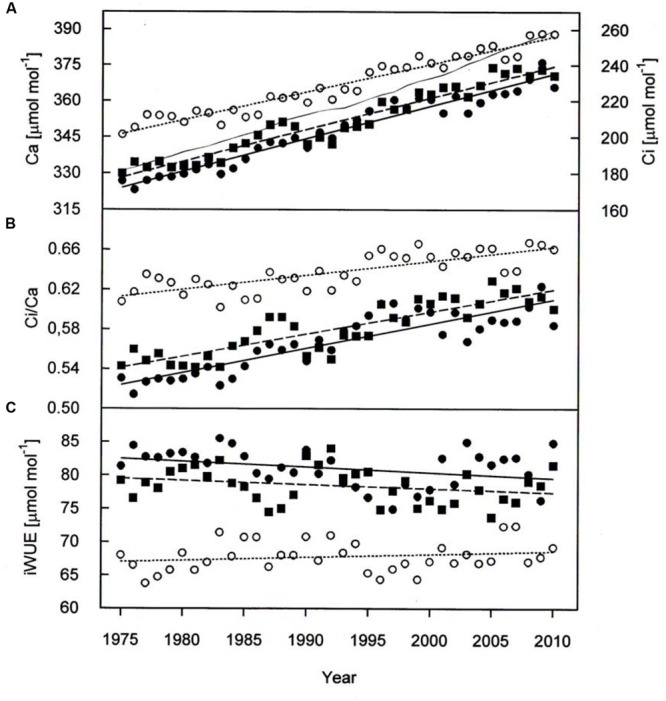
**(A)** Ambient armospheric CO_2_ concentration (*C_a_*, thin solid line) *C_i_*, **(B)** *C_i_*/*C_a_*, and **(C)** iWUE chronologies of *P. cembra* (solid circle, solid line), *P. abies* (solid square, dashed line) and *L. decidua* (open circle, dotted line) between 1975 and 2010. See **Table 2** for regression information.

Paralleling atmospheric CO_2_ enhancement (**Figure 4A**), tree ring derived *C_i_* increased from 1975 through 2010 from 180 to 234 μmol mol^-1^ in *P. cembra*, from 185 to 227 μmol mol^-1^ in *P. abies* and from 213 to 256 μmol mol^-1^ in *L. decidua* (**Figure 4A**; **Table 2**). Although species specific differences in the temporal change of *C_i_* were not statistically significant different from each other, mean *C_i_* was significantly lower in *P. cembra* and *P. abies* as compared to *L. decidua* (**Table 4**). Averaged over the study period *P. cembra* showed the lowest and *L. decidua*, the highest *C_i_*/*C_a_*, while the *C_i_*/*C_a_* of *P. abies* was intermediate (**Table 4**). The increase in *C_i_*/*C_a_* over time (**Figure 4B**; **Table 3**) was significantly higher in *P. cembra* and *P. abies* than in *L. decidua* (**Table 4**). In all the three species iWUE had remained stable during the study period (**Figure 4C**; **Table 3**). However, we observed statistically significant between species, with *P. cembra* showing the highest and *Larix decidua* showing the lowest iWUE averaged over the study period (**Table 4**).

### Effects *C_a_* and T_veg_ on *Δ^13^C, Δ^18^O*, BAI, *C_i_, C_i_*/*C_a_* and iWUE

Multiple linear regression analysis show that *Δ^13^C* and *Δ^18^O* of all species significantly increased with increasing ambient CO_2_ concentration (*C_a_*) while growing season mean air temperature (T_veg_) had no effect on *Δ^13^C* and on *Δ^18^O* (**Table 5**). Growth (BAI) of *P. cembra*, and *P. abies* significantly increased with increasing *C_a_* and T_veg_. *L. decidua* presented a significant increase in BAI at increasing *C_a_* without any response to T_veg_ (**Table 5**). For all species we found a significant increase in *C_i_* and *C_i_*/*C_a_* at higher *C_a_* but not at higher T_veg_ (**Table 5**). iWUE of *P. cembra, P. abies*, and *L. decidua*, however, did not significantly respond to increasing *C_a_* and T_veg_ (**Table 5**).

**Table 5:** Summary of multiple linear regression models fitted to explain inter-annual changes (1975–2010) in *Δ^13^C, Δ^18^O*, BAI, *C_i_, C_i_/*C*_a_*, and iWUE of *P. cembra, P. abies*, and *L. decidua* in response to atmospheric CO_2_ concentration (*C_a_*) and mean growing season (May-Sep) air temperature (T_veg_).

Species	Variable	coefficient	*SE*	β	*t*-value	*r*-value partial	*P*-value
***Δ^13^C***							
*P. cembra*	Intercept ***C_a_*** T_veg_	5.483 0.034- 0.058	1.225 0.004 0.067	0.90 3-0.091	4.477 .584 -0.865	0.831 0.149	<0.001 <0.001 0.393
*P. abies*	Intercept ***C_a_*** T_veg_	6.797 0.031 -0.064	1.054 0.003 0.058	0.925 -0.111	6.446 9.187 -1.106	0.848 -0.189	<0.001 <0.001 0.277
*L. decidua*	Intercept ***C_a_*** T_veg_	11.883 0.023 -0.164	1.027 0.003 0.056	0.901 -0.378	11.572 6.917 -2.900	0.769 -0.451	<0.001 <0.001 0.007
***Δ^18^O***							
*P. cembra*	Intercept ***C_a_*** T_veg_	49.624 -0.039 0.054	2.360 0.008 0.130	-0.742 0.060	21.030 -5.158 0.419	-0.668 0.073	<0.001 <0.001 0.678
*P. abies*	Intercept ***C_a_*** T_veg_	51.708 -0.050 0.143	2.363 0.008 0.130	-0.842 0.143	21.886 -6.513 1.0103	-0.750 0.189	<0.001 <0.001 0.278
*L. decidua*	Intercept ***C_a_*** T_veg_	45.863 -0.026 -0.094	2.219 0.007 0.122	-0.574 -0.120	20.671 -3.671 -0.768	-0.538 -0.0132	<0.001 0.001 0.448
**BAI**							
*P. cembra*	Intercept ***C_a_*** ***T_veg_***	-19.964 0.079 0.357	5.482 0.018 0.301	0.627 0.166	-3.642 4.472 1.184	0.614 0.202	0.001 <0.001 <0.001
*P. abies*	Intercept ***C_a_*** ***T_veg_***	-40.583 0.120 0.401	2.643 0.009 0.145	0.855 0.169	-15.354 13.990 2.761	0.925 0.433	<0.001 <0.001 0.009
*L. decidua*	Intercept ***C_a_*** T_veg_	-53.532 0.159 0.272	5.382 0.017 0.296	0.844 0.085	-9.947 9.128 0.919	0.846 0.158	<0.001 <0.001 0.365
***C_i_***							
*P. cembra*	Intercept ***C_a_*** T_veg_	-188.830 1.119 -1.033	19.045 0.062 1.047	0.991 -0.054	-9.915 18.130 -0.987	0.953 -0.169	<0.001 <0.001 0.331
*P. abies*	Intercept ***C_a_*** T_veg_	-177.500 1.100 -1.014	17.308 0.056 0.952	0.996 -0.054	-10.255 19.623 -1.065	0.960 -0.182	<0.001 <0.001 0.294
*L. decidua*	Intercept ***C_a_*** T_veg_	-113.938 0.987 -1.313	14.692 0.048 0.808	1.011 -0.079	-7.755 20.726 -1.625	0.964 -0.272	<0.001 <0.001 0.114
***C_i_/C_a_***							
*P. cembra*	Intercept ***C_a_*** T_veg_	0.037 0.002 -0.003	0.054 0.000 0.003	0.906 -0.087	0.680 8.752 -0.843	0.836 -0.145	0.501 <0.001 0.405
*P. abies*	Intercept ***C_a_*** T_veg_	0.087 0.001 -0.003	0.049 0.000 0.003	0.918 -0.096	1.795 9.118 -0.995	0.864 -0.164	0.082 <0.001 0.347
*L decidua*	Intercept ***C_a_*** T_veg_	0.321 0.001 -0.004	0.041 0.000 0.002	0.887 -0.0193	7.817 7.240 -1.574	0.783 -0.264	<0.001 <0.001 0.125
***iWUE***							
*P. cembra*	Intercept ***C_a_*** *T_veg_*	98.731 -0.062 0.534	9.964 0.032 0.548	-0.372 0.189	9.909 -1.915 0.974	-0.316 0.161	<0.001 0.064 0.337
*P. abies*	Intercept *C_a_* *T_veg_*	92.903 -0.053 0.534	9.052 0.029 0.534	-0.351 0.210	10.263 -1.798 1.072	-0.299 0.184	<0.001 0.081 0.291
*L. decidua*	Intercept *C****_a_*** T_veg_	59.644 0.007 0.695	7.661 0.025 0.421	0.052 0.317	7.785 0.270 1.650	0.047 0.276	<0.001 0.789 0.108

*Explanatory variables significantly influencing *Δ^*13*^C, Δ^*18*^O*, BAI, *C_*i*_, C_*i*_/*C*_a_*, and iWUE at *P* < 0.05 are in bolt and italics. Multiple linear regressions to obtain a relationships between (dependent) tree-ring variables and the explanatory variables *C_*a*_* and *T_*veg*_* were calculated according to: Tree-ring variable = a + (b^∗^*C_*a*_*) + (c^∗^T_*veg*_), where a, b, and c are fitting coefficients. Note that the *β*-coefficient expresses the relative importance of each explanatory variable in standardized terms. The direction of the relationships between variables (plus or minus) of the *β*-coefficients and the Pearson’s correlation coefficient indicates whether the relationship between the explanatory variable and the dependent variable is positive or negative.*

### Δ*^13^C*, Δ*^18^O*, and BAI Response to Climate (Climate-Growth Relationships)

**FIGURE 5:**
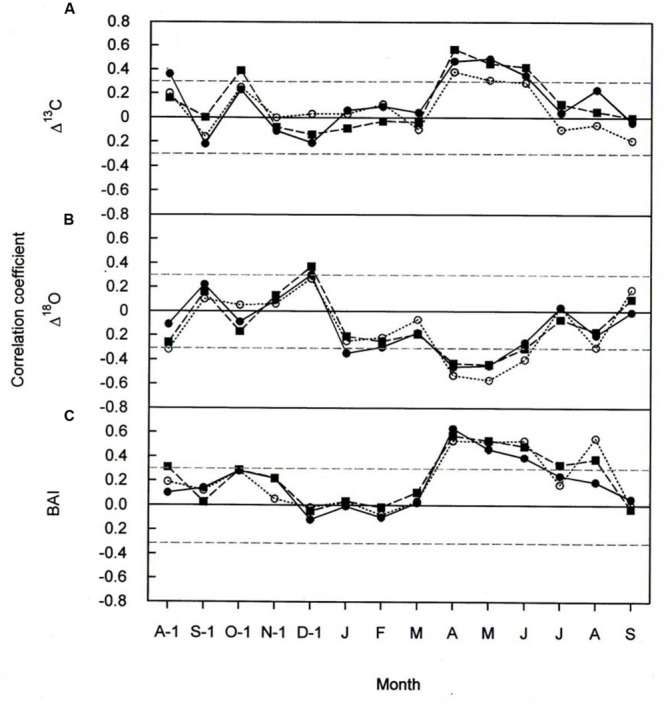
**Pearson correlation coefficients between **(A)***Δ^13^C***(B)***Δ^18^O*, and **(C)** BAI, and monthly mean temperature for *P. cembra* (solid circle, solid line), *P. abies* (solid square, dashed line) and *L. decidua* (open circle, dotted line) between 1975 and 2010.** Horizontal gray lines indicates *P* < 0.05.

**FIGURE 6:**
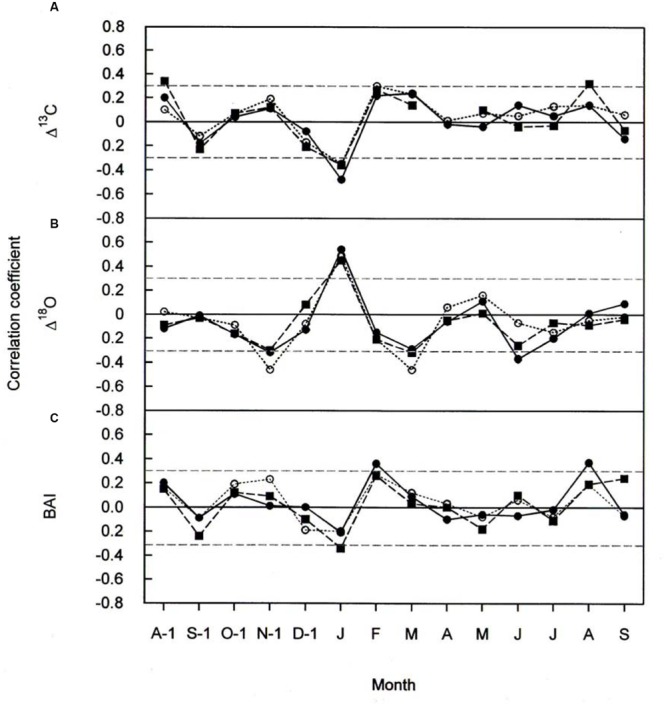
**Pearson correlation coefficients between **(A)***Δ^13^C***(B)***Δ^18^O*, and **(C)** BAI, and monthly total precipitation for *P. cembra* (solid circle, solid line), *P. abies* (solid square, dashed line) and *L. decidua* (open circle, dotted line) between 1975 and 2010.** Horizontal gray lines indicates *P* < 0.05.

Climate-response relationships of *Δ^13^C, Δ^18^O*, and BAI differed both in time and in signal strength (**Figures 5** and **6**). In all three species *Δ^13^C* was significantly positive correlated with April throughout June temperatures (**Figure 5A**) and significantly negative correlated with January precipitation (**Figure 6A**). Previous-year August and October temperature also favored *Δ^13^C* in *P. cembra* and in *P. abies*, respectively (**Figure 5A**), whereas previous- and current-year August precipitation did so in *P. abies* (**Figure 6A**).

The effects of temperature and precipitation on *Δ^18^O* were clearly in opposite directions (**Figures 5B** and **6B**). In all the three species tree-ring *Δ^18^O* was negative correlated to air temperature from April to June of the current year and significantly positive correlated to previous-year December temperature (**Figure 5B**) as well as to January precipitation (**Figure 6B**). From **Figure 5B**, previous- year August temperature had a negative correlation with *L. decidua* and current-year January temperature showed a negative correlation with *L. decidua*. Previous-year November and current-year March precipitation showed a negative correlation with *Δ^18^O* in *L. decidua*, as did June precipitation in *P. cembra* (**Figure 6B**).

We also found significant positive correlations between BAI and temperature during April, May, and June in *P. cembra, P. abies*, and *L. decidua* (**Figure 5C**). Current-year August temperature also favored BAI in *L. decidua* and *P. abies*, and also previous-year October temperature in *P. abies* (**Figure 5C**). The correlations between BAI and precipitation were weak, except for significant positive correlations in February and current-year August in *P. cembra* and a significant negative correlation in January in *P. abies* (**Figure 6C**).

## Discussion

Similar growth and *Δ^13^C*, and Δ*δ^18^O* responses were found over time in *P. cembra, P. abies* and *L. decidua* at the lower edge of the treeline ecotone in the central Austrian Alps. From 1975 throughout 2010 the three species increased *Δ^13^C* and BAI, while *Δ^18^O* showed a declining trend. Apparently, underlying response mechanisms were similar across the three studied species.

Our observed correlations for temperature and precipitation for *Δ^13^C* and *Δ^18^O* (**Figures 5** and **6**) are consistent with results reported for oak and pine trees at temperate sites in Switzerland (Saurer et al., 2008). Weather conditions prevailing during April through June predominantly were responsible for variations in tree-ring *Δ^13^C, Δ^18^O*, and BAI of *P. cembra, P. abies*, and *L. decidua*. We found positive correlations between April to June temperatures and *Δ^13^C*. *Δ^13^C* is strongly affected by net CO_2_ uptake rates, which at treeline are governed by both photon flux density and temperature (Treydte et al., 2001; McCarrol and Pawellek, 2004; Kress et al., 2011) as well as enhanced plant transpiration (Liu et al., 2015). Moreover, at our study site *in situ* net photosynthetic capacity of sun exposed twigs from the upper canopy of mature *P. cembra* and *L. decidua* trees measured under clear summer days also tended to increase between 1979 and 2007 (**Table 2**), which might be attributed to both the observed increase in atmospheric C_a_ and T_air_. Additionally, the temperature optimum of A_max_ for *P. cembra* increased from 12.5°C in 1956 (Pisek et al., 1969, 1973) to 15.0°C in 2002 (Wieser, 2004) and to 17.1°C in 2007 (Wieser et al., 2010), matching the observed increase in mean growing season air temperature of 0.9°C per decade (**Figure 2**). An increase in net photosynthetic rates under elevated CO_2_ was also observed in *P. mugo* and *L. decidua* after nine years of free-air CO_2_ enrichment at the Swiss treeline (Dawes et al., 2013; Streit et al., 2014). Three years of ecosystem warming also increased carbon uptake of *Pinus cembra* at treeline in the Austrian Alps (Wieser et al., 2015).

Our precipitation signals suggest that trees do not suffer from moisture stress. Indeed the observed declining trend *Δ^18^O* and the strong negative correlations between *Δ^18^O* and growing season precipitation is consistent with the physiological isotopic responses (Barbour and Farquhar, 2000), suggesting that stomatal conductance is increased during the study period. Although a leaf physiological signal in *δ^18^O* will be dampened at the level of tree rings due to oxygen exchange with source water during cellulose biosynthesis, impact of gw on ^18^O in tree rings may be still detectable, even in whole wood analyses (Weigt et al., 2015). Ecosystem warming accompanied by unchanged VPD also increased *g_w_* and hence also transpiration in boreal *Picea abies* (Bergh and Linder, 1999), *Pinus sylvestris* (Kellomäki and Wang, 1998), *Picea mariana* (Van Herk et al., 2011), *Pinus cembra* at treeline (Wieser et al., 2015), and *Populus deltoides* (Barron-Gafford et al., 2007). Thus, it seems that in cold environments under non-limiting water availability increasing temperatures counteract the diminishing effect of rising CO_2_ on leaf conductance (Saurer et al., 2014).

The observed positive correlations between BAI and April–June temperatures are also reflected in wood formation. At the study site wood formation of larch, spruce and pine generally starts in May, reaches its maximum in June, and terminates in August (Havranek, 1981; Loris, 1981; Gruber et al., 2009). Beside summer temperatures (**Figure 5C**) other climatic variable like winter and August precipitation (**Figure 6C**) are also known to influence radial growth at treeline as shown for *P. cembra* by Oberhuber (2004), reflecting minor soil water effects on tree growth at treeline (Tranquillini, 1979; Wieser, 2004). Although treeline trees are saturated with carbohydrates (Gruber et al., 2011), growth of trees at treeline is primarily affected by temperature dependent carbon sink activity during tissue formation (Hoch and Körner, 2003, 2012). The observed increase in BAI (**Figure 3C**) suggests that our treeline trees benefit from climate warming, although effects of CO_2_ fertilization on growth may not completely ruled out (**Table 5**). Four years of experimental air warming with open-top-chambers also stimulated radial growth *of Picea glauca* seedlings at the subarctic treeline in southwest Yukon, Canada (Danby and Hik, 2007). Thus, when growth is stimulated and there is plenty of water *g_w_* can increase as indicated by a decline in *Δ^18^O* (**Figure 3B**) along with increasing *A*, resulting in a constant iWUE (**Figure 4C**).

Elevated atmospheric CO_2_ is expected to affect plant carbon-water relationships, as a decline in stomatal conductance is often observed when plants are exposed to elevated CO_2_ (Battipaglia et al., 2013). If stomatal conductance declines under increasing CO_2_ in combination with an increased or unchanged carbon assimilation, this will decrease the *C_i_* to *C_a_* ratio and thus decrease *Δ^13^C*. Conversely, in all three study species *Δ^13^C* increased from 1975 throughout 2010, while tree-ring derived iWUE remained stable (**Figure 4C**) although ambient CO_2_ concentration increased by 60 μmol mol^-1^ (**Figure 2B**). The stability of iWUE resulted as *C_i_* drifted upward paralleling the rise in *C_a_* (**Figure 2B**). Likewise, in *Picea schrenkiana* at treeline in the western Tianshan Mountains in China iWUE remained also unchanged from 1985 to 2010 (Wu et al., 2015). No change in iWUE (i.e., homeostasis) over the last 100 years was also reported for three conifer species in the Selkirk Range (Rocky Mountains, Idaho, ID, USA) by Marshall and Monserud (1996). Other studies by contrast observed a 20% increase in iWUE from the 1960 throughout 2000 in mature trees in tropical, arid, Mediterranean, wet temperate and boreal forests distributed through Europe, Asia, Africa, America, and Oceania (Penuelas et al., 2011; Saurer et al., 2014; Frank et al., 2015). In these latter studies, increasing iWUE was attributed to the combined effect of increasing CO_2_ and climate change-induced soil drying that reduced stomatal aperture. Soil drought can be ruled out along the treeline ecotone of the Central Alps (Mayr, 2007; Wieser, 2012). Occurrence of soil drought strongly depends on site conditions such as precipitation patterns, water holding capacity of the soil, and evaporative demand. Ample precipitation and moderate evaporative demand in general cause soil water availability to be sufficiently high to meet the trees’ water demand at treeline in the Central Tyrolean Alps (Mayr, 2007; Wieser et al., 2009). As a consequence, treeline trees are rarely forced to restrict transpiration (Tranquillini, 1979; Benecke et al., 1981; Matyssek et al., 2009; Wieser and Leo, 2012; Wieser et al., 2014, 2015). Given the ample soil water availability whole-tree conductance of *P. cembra, P. abies*, and *L. decidua* remains high for CO_2_ uptake because leaf conductance for water vapor depends only on the evaporative demand driven by irradiance and vapor pressure deficit (Wieser, 2012).

Beside climate warming and increasing *C_a_*, nitrogen deposition could also be important for explaining the observed increase in tree growth as increasing nitrogen deposition during the 1980ties (Smidt and Mutsch, 1993) has been suggested as a possible growth stimulator. However, there is evidence that nitrogen contents per needle dry mass are higher in trees at treeline as compared to trees growing at lower elevation sites (Körner, 1989; Birmann and Körner, 2009). Furthermore, since 1988, nitrogen deposition at treeline in the Tyrolean Alps is steadily declining (Amt der Tiroler Landesregierung, 2015), and a nitrogen fertilizer experiment at the alpine treeline in the Swiss Alps showed little or no growth stimulation (Keller, 1970). Thus, it seems that presently nitrogen deposition is insufficient to explain observed growth trends at treeline as reported previously by Tranquillini (1979) and Nicolussi et al. (1995).

## Conclusion

Treeline trees respond to the increasing atmospheric CO_2_ level (*C_a_*) in a way that their leaf-intercellular CO_2_ concentration (*C_i_*) drifted upward paralleling the rise in atmospheric CO_2_ while iWUE remained stable over the last 36 years. The stability in iWUE was accompanied by an increase in BAI suggesting that treeline trees benefit from both recent climate warming) and CO_2_ fertilization. In addition, treeline trees are rarely forced to restrict transpiration due to ample soil water availability (Tranquillini, 1979; Matyssek et al., 2009; Wieser et al., 2015). A stable iWUE suggests an increase of both carbon gain and leaf conductance for water vapor as also indicated by stable C and O isotope analysis and direct gas exchange assessments. Furthermore, iWUE may not change species composition at treeline in the Austrian Alps due to similar ecophysiological responses to climatic changes of the three sympatric study species. Our finding that growth of treeline associated conifers increases with slowly rising ambient CO_2_ concentration and warming may be relevant for assessing complex growth models with empirical data, finally leading to model improvements and better estimations of forest-climate feedbacks.

## Author Contributions

GW, RM, and TG conceived and designed the experiment. WO, AG, and ML performed the experiment. GW, WO, AG, and ML analyzed the data. GW, RM, and TG wrote the manuscript and WO and AG provided editorial advice.

## Conflict of Interest Statement

The authors declare that the research was conducted in the absence of any commercial or financial relationships that could be construed as a potential conflict of interest.

## Footnotes

1 www.scrippsco2.ucsd.edu/home

2 http://cdiac.ornl.gov/trends/co2/sio-mlo.html

## References

[B1] Amt der Tiroler Landesregierung (2015). *Luftgüte in Tirol. Bericht über das Jahr 2014*. Innsbruck: Amt der Tiroler Landesregierung.

[B2] Barbour M. M. (2007). Stable oxygen isotope composition of plant tissue: a review. *Funct. Plant Biol.* 34 83–94. 10.1071/FP06228 32689335

[B3] Barbour M. M. Farquhar G. D. (2000). relative humidity and ABA-induced variation in carbon and oxygen isotope ratios of cotton leaves. *Plant Cell Environ.* 23 473–485. 10.1046/j.1365-3040.2000.00575.x

[B4] Barbour M. M. Fischer R. A. Sayre K. D. Farquhar G. D. (2000). Oxygen isotope ratio of leaf and grain material correlates with stomatal conductance and grain yield in irrigated wheat. *Austr. J. Plant Physiol.* 27 625–637.

[B5] Barron-Gafford G. A. Grieve K. A. Murthy R. (2007). Leal-and stand-level responses of a forested mesocosm to independent manipulations of temperature and vapour pressure deficit. *New Phytol.* 174 614–625. 10.1111/j.1469-8137.2007.02035.x 17447916

[B6] Battipaglia G. Saurer M. Cherubini P. Calfapietra C. McCarthy H. R. Norby R. J. (2013). Elevated CO_2_ increases tree-level intrinsic water use efficiency: insights from carbon and oxygen isotope analyses in tree rings across three forest FACE sites. *New Phytol.* 197 544–554. 10.1111/nph.12044 23215904

[B7] Benecke U. Schulze E.-D. Matyssek R. Havranek W. M. (1981). Environmental control of CO_2_-assimilation and leaf conductance in *Larix decidua* Mill. I. a comparison of contrasting natural environmets. *Oecologia* 50 54–61. 10.1007/BF00378793 28310061

[B8] Bergh J. Linder S. (1999). Effects of soil warming during spring on photosynthetic recovery in boreal Norway spruce stands. *Glob. Change Biol.* 5 245–253. 10.1046/j.1365-2486.1999.00205.x

[B9] Birmann K. Körner C. (2009). Nitrogen status of conifer needles at the alpine treeline. *Plant Ecol. Divers.* 2 233–241. 10.1080/17550870903473894

[B10] Borella S. Leuenberger M. Saurer M. Siegwolf R. (1998). Reducing uncertainties in δ13C analysis of tree rings: pooling, milling and cellulose extraction. *J. Geophys. Res.* 103 519–526. 10.1029/98JD01169

[B11] Brendel O. Iannetta P. P. M. Stewart D. (2000). Arapid and simple method to isolate pure alpha-cellulose. *Phytochem. Anal.* 11 7–10. 10.1002/(SICI)1099-1565(200001/02)11:1<7::AID-PCA488>3.0.CO;2-U

[B12] Brugnoli E. Farquhar G. D. (2000). “Photosynthetic fractionation of carbon isotopes,” in *Photosynthesis: Physiology and Metabolism* Vol. 9 eds Leegood R. C. Sharkey T. D. von Caemmerer S. (Dordrecht: Kluwer Academic Publishers) 399–434.

[B13] Bunn A. G. Graumlich L. J. Urban D. L. (2005). Trends in twentieth century tree growth at high elevations in the Sierra Nevada and White Mountains, USA. *Holocene* 15 481–488. 10.1191/0959683605hl827rp

[B14] Büntgen U. Esper J. Frank D. C. Nicolussi K. Schmidhalter M. (2005). A 1052-year tree-ring proxy for Alpine summer temperature. *Clim. Dyn.* 25 141–153. 10.1007/s00382-005-0028-1

[B15] Büntgen U. Frank D. C. Nievergelt D. Esper J. (2006). Summer temperature variations in the European Alps, AD 755–2004. *J. Clim.* 19 5606–5623. 10.1073/pnas.1211485110

[B16] Carrer M. Urbinati C. (2004). Age-dependent tree-ring growth response to climate in *Larix decidua* and *Pinus cembra*. *Ecology* 85 730–740. 10.1890/02-0478

[B17] Carrer M. Urbinati C. (2006). Long-term change in the sensitivity of tree-ring growth to climate forcing in *Larix decidua*. *New Phytol.* 170 861–872. 10.1111/j.1469-8137.2006.01703.x 16684244

[B18] Cernusak L. A. Ubierna N. Winter K. Holtum J. A. M. Marshall J. D. Farquhar G. D. (2013). Environmental and physiological determinants of carbon isotope discrimination in terrestrial plants. *New Phytol.* 200 950–965. 10.1111/nph.12423 23902460

[B19] Danby R. K. Hik D. S. (2007). Response of white spruce (*Pinus glauca*) to experimental warming at a subarctic alpine treeline. *Glob. Change Biol.* 13 437–451. 10.1111/j.1365-2486.2006.01302.x

[B20] Dawes M. A. Hagedorn F. Handa I. T. Streit K. Ekbald Q. A. Rixen C. (2013). An alpine treeline in a carbon dioxide-rich world: synthesis of a nine-year free-air carbon dioxide enrichment study. *Oecologia* 171 623–637. 10.1007/s00442-012-2576-5 23340765

[B21] Duquesnay A. Breda N. Stievenard M. Dupouey J. L. (1998). Changes of tree-ring δ13C and water-use efficiency of beech (*Fagus sylvatica* L.) in north-eastern France during the past century. *Plant Cell Environ.* 21 565–572. 10.1046/j.1365-3040.1998.00304.x

[B22] Farquhar G. D. Ehleringer J. R. Hubick K. T. (1989). Carbon isotope discrimination and photosynthesis. *Annu. Rev. Plant Physiol.* 40 503–537. 10.1146/annurev.pp.40.060189.002443

[B23] Farquhar G. D. O’Leary M. H. Berry J. A. (1982). On the relationship between carbon isotope discrimination and the intercellular carbon dioxide concentration in leaves. *Aust. J. Plant. Physiol.* 9 121–137. 10.1071/pp9820121

[B24] Farquhar G. D. Richards R. A. (1984). Isotopic composition of plant carbon correlates with water-use efficiency of wheat genotype. *Aust. J. Plant. Physiol.* 11 539–552. 10.1071/PP9840539

[B25] Flexas J. Ribas-Carbo M. Diaz-Espejo A. Glamés J. Medrano H. (2008). Mesophyll conductance to CO_2_: current knowledge and future prospects. *Plant Cell Environ.* 31 602–621. 10.1111/j.1365-3040.2007.01757.x 17996013

[B26] Frank D. Esper J. (2005). Temperature reconstructions and comparisons with instrumental data from a tree-ring network for the European Alps. *Int. J. Climatol.* 25 1437–1454. 10.1002/joc.1210

[B27] Frank D. C. Poulter B. Saurer M. Esper J. Huntingford C. Helle G. (2015). Water-use efficiency and transpiration across European forests during the Anthropocene. *Nat. Clim. Change* 5 579–583. 10.1038/NCLIMATE2614

[B28] Fritts H. C. (1976). *Tree Rings and Climate.* London: Academic Presss.

[B29] Gessler A. Brandes E. Keitel C. Boda S. Kayler Z. Granier A. (2013). The oxygen isotope enrichment of leaf-exported assimilates—does it always reflect lamina leaf water enrichment? *New Phytol.* 200 144–157. 10.1111/nph.12359 23763637 PMC3902987

[B30] Grams T. E. E. Kozovits A. R. Häberle K.-H. Matyssek R. Dawson T. E. (2007). Combining δ13C and δ18O analyses to unravel competition, CO_2_ and O_3_ effects on the physiological performance of different-aged trees. *Plant Cell Environ.* 30 1023–1034. 10.1111/j.1365-3040.2007.01696.x 17617829

[B31] Graumlich L. J. (1991). Subalpine tree growth, climate, and increasing CO_2_: an assessment of recent growth trends. *Ecology* 72 1–11. 10.2307/1938895

[B32] Graumlich L. J. Brubaker L. B. Grier C. C. (1989). Long-term growth trends in forest net primary productivity: cascade Mountains, Washington. *Ecology* 70 405–410. 10.2307/1937545

[B33] Grissino-Mayer H. D. (2001). Evaluating crossdating accuracy: a manual and tutorial for the computer program COFECHA. *Tree Ring Res.* 57 205–221.

[B34] Gruber A. Pirkebner D. Oberhuber W. Wieser G. (2011). Spatial and seasonal variations in mobile carbohydrates in *Pinus cembra* in the timberline ecotone of the Central Austrian Alps. *Eur. J. Forest Res.* 130 173–179. 10.1007/s10342-010-0419-7 PMC3191523 22003357

[B35] Gruber A. Wieser G. Oberhuber A. (2009). Intra-annual dynamics in stem CO2 eﬄux in relation to cambial activity and xylem development in *Pinus cembra*. *Tree Physiol.* 29 641–649. 10.1093/treephys/tpp001 19203979 PMC3013296

[B36] Havranek W. M. (1981). Stammatmung, Dickenwachstum und Photosynthese einer Zirbe (*Pinus cembra*) an der Waldgrenze. *Mitt Forstl. Bundesvers Wien.* 142 443–467.

[B37] Hoch G. Körner C. (2003). The carbon charging of pines at the climatic treeline: a global comparison. *Oecologia* 135 10–21. 10.1007/s00442-002-1154-7 12647099

[B38] Hoch G. Körner C. (2012). Global patterns of mobie carbon stores in trees at the high-elevation tree line. *Glob. Ecol. Biogeogr.* 21 861–871. 10.1111/j.1466-8238.2011.00731.x

[B39] Holmes R. L. (1994). *Dendrochronology Program Library User’s Manual. Laboratory of Tree-Ring* Tucson: Research University of Arizona.

[B40] Holtmeier F.-K. Broll G. (2007). Treeline advance – driving processes and adverse factors. *Landscape* 1 1–33.

[B41] Jacoby G. C. D’Arrigo R. D. (1997). Tree rings, carbon dioxide, and climate change. *Proc. Natl. Acad. Sci. U.S.A.* 94 8350–8353. 10.1073/pnas.94.16.8350 11607744 PMC33752

[B42] Jaggi M. Saurer M. Fuhrer J. Siegwolf R. (2002). The relationship between the stable carbon isotope composition of needle bulk material, starch, and tree rings in *Picea abies*. *Oecologia* 131 325–332. 10.1007/s00442-002-0881-0 28547703

[B43] Keller T. (1970). Wuchsleistung, Gaswechsel, Überlebensprozente und Schnee-schimmelpilzbefall gedüngter Ballenpflanzen an der oberen Waldgrenze. *Mitt. Schweiz. Anstalt Forstl. Versuchsw.* 46 1–32.

[B44] Kellomäki S. Wang K.-Y. (1998). Sap flow in *Scots pines* growing under conditions of year-round carbon dioxide enrichment and temperature elevation. *Plant Cell Environ.* 21 969–981. 10.1046/j.1365-3040.1998.00352.x

[B45] Körner C. (1989). The nutrient status of plants from high altitude. A world-wide comparison. *Oecologia* 81 379–391. 10.1007/BF00377088 28311193

[B46] Kress A. Saurer M. Siegwolf R. T. W. Frank D. Esper J. Bugmann H. (2011). A 350 year drought reconstruction from Alpine tree ring stable isotopes. *Global Biogeochem. Cycles* 24 10.1029/2009GB003613

[B47] Kunter M. Nachtsheim C. Neter J. Li W. (2004). *Applied Linear Statistical Models* 5th Edn Irwin: McGraw-Hill.

[B48] Larcher W. (2001). *Ökophysiologie der Pflanzen: Leben, Leistung und Stressbewältigung der Pflanzen in ihrer Umwelt.* Stuttgart: Ulmer.

[B49] Levesque M. Siegwolf R. Saurer M. Eilmann B. Brang P. Bugmann H. (2014). Drought response of five conifer species under contrasting water availability suggests high vulnerability of Norway spruce and European larch. *Glob. Change Biol.* 19 3184–3199. 10.1111/gcb.12268 23712589

[B50] Liu X. Wang W. Xu G. Zeng X. Wu G. Zhang X. (2015). Tree growth and intrinsic water-use efficiency of inland riparuian forests in norhwestern China: evaluation via δ13C and δ18O analysis of tree rings. *Tree Physiol.* 34 966–980. 10.1093/treephys/tpu067 25145697

[B51] Loader N. J. McCarrol D. Gagen M. Robertson I. Jalkanen R. (2007). “Extracting climatic information from stable isotopes in tree rings,” in *Stable Isotopes as Indicators of Ecological Change* eds Dawson T. E. Siegwolf R. T. W. (London: Academic Press) 27–48.

[B52] Loris K. (1981). Dickenwachstum von Zirbe, Fichte und Lärche an der alpinen Waldgrenze/Patscherkofel. Ergebnisse der Dendrometermessungen 1976/79. *Mitt. Forstl. Bundesvers. Wien.* 142 417–441.

[B53] Marshall J. D. Monserud R. A. (1996). Homeostatic gas-exchange parameters inferred from 13C/12C in tree rings of conifers. *Oecologia* 105 13–21. 10.1007/BF00328786 28307117

[B54] Matyssek R. Wieser G. Patzner K. Blaschke H. Häberle K.-H. (2009). Transpiration of forest trees and stands at different altitude: consistencies rather than contrasts? *Eur. J. Forest Res.* 128 579–596. 10.1007/s10342-008-0243-5

[B55] Mayr S. (2007). “Limits in water relations,” in *Trees at Their Upper Limit. Treelife Limitation at the Alpine Timberline* eds Wieser G. Tausz M. (Dordrecht: Springer) 145–162.

[B56] McCarrol D. Pawellek E. (2004). Stable carbon isotope ratios of *Pinus sylvestris* from northern Finland and the potential for extracting a climate signal from long term Fennoscandian chronologies. *Holocene* 11 517–526. 10.1191/095968301680223477

[B57] Neuwinger I. (1972). Standortuntersuchungen am Sonnberg im Sellrainer Obertal, Tirol. *Mitt. Forstl. Bundesvers. Wien.* 96 177–207.

[B58] Nicolussi K. Bortenschlager S. Körner C. (1995). Increase in tree-ring width in subalpine Pinus cembra from the centrak Alps may be CO_2_-related. *Trees* 9 181–189. 10.1007/BF00195270

[B59] Oberhuber W. (2004). Influence of climate on radial growth of *Pinus cembra* within the alpine timberline ecotone. *Tree Physiol.* 24 291–301. 10.1093/treephys/24.3.291 14704138

[B60] Oberhuber W. (2007). “Limitation by growth processes,” in *Trees at Their Upper Limit. Treelife Limitation at the Alpine Timberline* eds Wieser G. Tausz M. (Dordrecht: Springer) 131–143.

[B61] Oberhuber W. Kofler W. Pfeifer K. Seeber A. Gruber A. Wieser G. (2008). Long-term changes in tree-ring-climate relationships at Mt. Patscherkofel (Tyrol, Austria) since the mid 1980s. *Trees* 22 31–40. 10.1007/s00468-007-0166-7 21532976 PMC3083837

[B62] Penuelas J. Canadell J. G. Ogaya R. (2011). Increased water-use efficiency during the 20th century did not translate into enhanced tree growth. *Global Ecol. Biogeogr.* 20 597–608. 10.1111/j.1466-8238.2010.00608.x

[B63] Peterson D. L. Arbaugh M. J. Robinson L. J. Dederian B. I. (1990). Growth trends of whitebark pine and lodgepole pine in a subalpine Sierra Nevada forest, California, USA. *Arct. Alp. Res.* 22 233–243. 10.2307/1551586

[B64] Pilcher J. R. (1990). “Sample preparation, cross-dating and measurement,” in *Methods of Dendrochronology: Applications in the Environmental Sciences* eds Cook E. R. Kairiukstis L. A. (Dordrecht: Kluwer) 40–51.

[B65] Pisek A. Larcher W. Moser W. Pack I. (1969). Kardinale Temperaturbereiche der Photosynthese und Grenztemperaturen des Lebens der Blätter verschiedener Spermatophyten. III. Temperaturabhängigkeit und optimaler Temperaturbereich der Netto-Photosynthese. *Fora Ab. B* 158 608–630.

[B66] Pisek A. Larcher W. Vegis A. Napp-Zinn K. (1973). “The normal temperature range,” in *Temperature and Life* eds Precht H. Christophersen J. Hensel H. Larcher W. (Berlin: Springer) 102–194.

[B67] Poage M. A. Chamberlain C. P. (2001). Empirical relationships between elevation and stable isotope composition of precipitation and surface waters: considerations for studies of paleoelevation change. *Am. J. Sci.* 301 1–15. 10.2475/ajs.301.1.1

[B68] Roden J. S. Farquhar G. D. (2012). A controlled test of the dual-isotope approach for the interpretation of stable carbon and oxygen isotope ratio variation in tree rings. *Tree Physiol.* 32 490–503. 10.1093/treephys/tps019 22440882

[B69] Rolland C. Florence-Schueller J. (1998). Dendroclimatological synthesis on mountain pine (*Pinus uncinata* Mill. Ex Mirb.) in the Pyrenees and the Alps. *Ecology* 29 417–421.

[B70] Sarris D. Siegwolf R. Körner C. (2013). Inter and intra-annual stable carbon and oxygen isotope signals in response to drought in Mediterranean pines. *Agric. Forest Meteorol.* 168 59–68. 10.1016/j.agrformet.2012.08.007

[B71] Saurer M. Allen K. Siegwolf R. T. W. (1997). Correlating δ13C and δ18O in cellulose of trees. *Plant Cell Environ.* 20 1543–1550. 10.1046/j.1365-3040.1997.d01-53.x

[B72] Saurer M. Cherubini P. Reynolds-Henne C. E. Treydte K. S. Anderson W. T. Siegwolf R. T. W. (2008). An investigation of the common signal in tree ring stable isotope chronologies at temperate sites. *J. Geophys. Res.* 113:G 04035 10.1029/2008JG000689

[B73] Saurer M. Spahni R. Frank D. C. Joos F. Leuenberger M. Loader N. J. (2014). Spatial variability and temporal trends in water-se efficiency of European forest. *Glob. Change Biol.* 20 332–336. 10.1111/gcb.12717 25156251

[B74] Scheidegger Y. Saurer M. Bahn M. Siegwolf R. (2000). Linking stable isotopes with stomatal conductance and photosynthetic capacity: a conceptual model. *Oecologia* 125 350–357. 10.1007/s004420000466 28547329

[B75] Seibt U. Rajabi A. Griffiths H. Berry J. (2008). Carbon isotopes and water use efficiency: sense and sensitivity. *Oecologia* 155 441–454. 10.1007/s00442-007-0932-7 18224341

[B76] Sidorova O. V. Siegwolf R. T. W. Saurer M. Shashkin A. V. Knorre A. A. Prokushkin A. S. (2009). Do centennial tree-ring and stable isotope trends of *Larix gmelinii* (Rupr.) indicate increasing water shortage in the Siberian north? *Oecologia* 161 825–835. 10.1007/s00442-009-1411-0 19590897

[B77] Silva L. C. R. Anand M. (2013). Probing for the influence of atmospheric CO_2_ and climate change on forest ecosystems across biomes. *Glob. Ecol. Biogeogr.* 22 83–92. 10.1111/j.1466-8238.2012.00783.x

[B78] Smidt S. Mutsch F. (1993). “Messungen der nassen Freilanddeposition an alpinen Höhenprofilen,” in *Proceedings of the International Symposium ‘Stoffeinträge aus der Atmosphäre und Waldbodenbe-Lastung in der Ländern der ARGE ALP und ALPEN ADRIA’*. GSF-Report, Neuherberg 21–29.

[B79] Sohn J. A. Gebhardt T. Ammer C. Bauhus J. Häberle K.-H. Matyssek R. (2013). Mitigation of drought by thinning: short-term and long-term effects on growth and physiological performance of Norway spruce (*Picea abies*). *Forest Ecol. Manag.* 308 188–197. 10.1016/j.foreco.2013.07.048

[B80] Streit K. Siegwolf R. T. W. Hagedorn F. Schaub M. Buchmann N. (2014). Lack of photosynthetic or stomatal regulation after 9 years of elevated [CO_2_] and 4 years of soil warming in two conifer species at the alpine treeline. *Plant Cell Environ.* 37 315–326. 10.1111/pce.12197 24003840

[B81] Tranquillini W. (1979). *Physiological Ecology of the Alpine Timberline. Tree Existence in High Altitudes with Special Reference to the European Alps. Ecological Studies 31.* Berlin: Springer.

[B82] Treydte K. Boda S. Graf Pannatier E. Fonti P. Frank D. Ullrich B. (2014). Seasonal transfer of oxygen isotopes from precipitation and soil to the tree ring: source water versus needle water enrichment. *New Phytol.* 202 772–783. 10.111/nph.12741 24602089

[B83] Treydte K. S. Schleser G. H. Schweingruber F. H. Winiger M. (2001). The climatic significance of δ13C in subalpine spruces (Lötschental. Swiss Alps). *Tellus Ser. B* 55 593–611. 10.1034/j.1600-0889.2001.530505.x

[B84] Van Herk I. G. Gower S. T. Bronson D. R. Tanner M. S. (2011). Effect of climate warming on canopy water dynamics of a boreal black spruce plantation. *Can. J. For. Res.* 41 217–227. 10.1139/X10-196

[B85] Volgger E. (1995). *Zur Ozonempfindlichkeit der Europäischen Lärche (Larix decidua Mill.) an der Waldgrenze.* Diploma thesis, Botany, University of Innsbruck Innsbruck.

[B86] Weigt R. B. Bräunlich S. Zimmermann L. Saurer M. Grams T. E. E. Dietrich H.-P. (2015). Comparison of δ13C and δ18O values between tree-ring whole wood and cellulose in five species growing under two different site conditions. *Rapid Commun. Mass Spectr.* 29 2233–2244. 10.1002/rcm.7388 26522315

[B87] Werner C. Schnyder H. Cuntz M. Keitel C. Zeeman M. J. Dawson T. E. (2012). Progress and challenges in using stable isotopes to trace plant carbon and water relations across scales. *Biogeosciences* 9 3083–3111. 10.5194/bg-9-3083-2012

[B88] Wieser G. (2004). Environmental control of carbon dioxide gas exchange in needles of a mature Pinus cembra tree at the alpine timberline during the growing season. *Phyton* 44 145–153.

[B89] Wieser G. (2012). Lessons from the timberline ecotone in the Central Tyrolean Alps: a review. *Plant Ecol. Div.* 5 127–139. 10.1080/17550874.2010.498062

[B90] Wieser G. Gigele T. Pausch H. (2005). Seasonal and spatial variation of woody tissue respiration in a *Pinus cembra* tree at the alpine timberline in the Central European Alps. *Eur. J. For. Res.* 124 1–8. 10.1007/s10342-004-0050-6

[B91] Wieser G. Grams T. E. E. Matyssek R. Oberhuber W. Gruber A. (2015). Soil warming increased whole-tree water use of *Pinus cembra* at the treeline in the Central Tyrolean Alps. *Tree Physiol.* 35 279–288. 10.1093/treephys/tvp009 25737326 PMC4820648

[B92] Wieser G. Gruber A. Oberhuber W. (2014). Sap flow characteristics and whole-tree water use of *Pinus cembra* across the treeline ecotone in the central Tyrolean Alps. *Eur. J. For. Res.* 133 287–295. 10.1007/s10342-013-0760-8

[B93] Wieser G. Leo M. (2012). Whole tree water use by *Pinus cembra* at the treeline in the Central Tyrolean Alps. *Plant Ecol. Div.* 5 81–88. 10.1093/treephys/tpv009

[B94] Wieser G. Matyssek R. Luzian R. Zwerger P. Pindur P. Oberhuber W. (2009). Effects of atmospheric and climate change at the timberline of the Central European Alps. *Ann. For. Sci.* 66:402 10.1051/forest/2009023 PMC3047780 21379395

[B95] Wieser G. Oberhuber W. Walder L. Spieler D. Gruber A. (2010). Photosynthetic temperature adaptation of *Pinus cembra* within the timberline ecotone of the Central Austrian Alps. *Ann. For. Sci.* 67:201 10.1051/forest/2009094 PMC3047779 21379394

[B96] Wu G. Liu X. Chen T. Xu G. Wang W. Zeng X. (2015). Elevation-dependent variations of tree growth and intrinsic water-use efficiency in Schrenk spruce (*Picea schrenkiana*) in the westen Tianshan Mountain, China. *Front. Plant Sci.* 6:309 10.3389/fpls.2015.00309 PMC4422019 25999973

[B97] Zar J. H. (1999). *Biostatistical Analysis.* Upper Saddle River, NJ: Prentice Hall.

